# Autologous platelet concentrate augmentation in anterior cruciate ligament reconstruction: a level I evidence-based meta-analysis

**DOI:** 10.1007/s00132-025-04748-y

**Published:** 2025-11-28

**Authors:** Filippo Migliorini, Ludovico Lucenti, Luise Schäfer, Francesco Simeone, Gennaro Pipino, Naveen Jeyaraman, Madhan Jeyaraman

**Affiliations:** 1 Department of Trauma and Reconstructive Surgery, University Hospital of Halle, Martin-Luther University Halle-Wittenberg, Ernst-Grube-Street 40, 06097 Halle (Saale), Germany; 2 Department of Orthopaedic and Trauma Surgery, Academic Hospital of Bolzano (SABES-ASDAA), via Lorenz Böhler 5, 39100 Bolzano, Italy; 3 Department of Life Sciences, Health, and Health Professions, Link Campus University, Via del Casale di San Pio V, 00165 Rome, Italy; 4 Department of Orthopaedic and Trauma Surgery, Eifelklinik St, Brigida, 52152 Simmerath, Germany; 5 Department of Precision Medicine in Medical, Surgical and Critical Care (Me.Pre.C.C.), University of Palermo, 90133 Palermo, Italy; 6 Department of Orthopaedics, ACS Medical College and Hospital, Dr MGR Educational and Research Institute, 600077 Chennai, Tamil Nadu India; 7 Department of Regenerative Medicine, Agathisha Institute of Stemcell and Regenerative Medicine (AISRM), 600030 Chennai, Tamil Nadu India; 8 Department of Orthopaedics, Villa Erbosa Hospital, Bologna, Italy

**Keywords:** Anterior cruciate ligament tears, Anterior cruciate ligament reconstruction, Autologous platelet concentrate, Platelet-rich plasma, Patient-related outcome measures, Vordere Kreuzbandruptur, Vordere Kreuzbandrekonstruktion, Autologe Thrombozytenkonzentrate, Plättchenreiches Plasma, Patient-Related Outcome Measures

## Abstract

**Introduction:**

Anterior cruciate ligament (ACL) injuries are common in young active individuals and reconstruction remains the standard treatment. Recently, biological augmentation with autologous platelet concentrates (APC) has gained interest given the potential to enhance graft healing. This meta-analysis examined whether the use of APC improves patient-reported outcome measures (PROM) and reduces complications after ACL reconstruction.

**Methods:**

Randomized controlled trials (RCT) comparing standard ACL reconstruction with APC-augmented procedures were included. Eligible articles were peer-reviewed and published in English, German, Italian, French or Spanish. A systematic search of PubMed, Web of Science and Embase was performed in June 2025. Extracted data included demographics, follow-up and PROMs. Methodological quality was assessed using the Cochrane RoB2 tool. Meta-analyses were conducted with Review Manager 5.3, applying inverse variance and Mantel-Haenszel methods. A subgroup analysis was performed for platelet-rich plasma (PRP).

**Results:**

A total of 14 RCTs comprising 869 patients met the inclusion criteria. Methodological quality was acceptable, with no study rated as having a high risk of bias. The mean follow-up was 12.2 ± 7.4 months. Baseline features and PROMs were comparable between groups. The use of APC augmentation significantly reduced anterior tibial laxity but the effect size was below the clinical threshold. No significant differences were observed in PROMs or revision rates.

**Conclusion:**

The current level of I evidence does not support APC augmentation as a means to improve short-term outcomes or reduce complications after ACL reconstruction; however, its safety profile and biological rationale justify further high-quality RCTs with standardized protocols and longer follow-up.

**Supplementary Information:**

The online version of this article (https://doi.org/10.1007/s00132-025-04748-y) contains supplementary material, which is available to authorized users.

## Abstract (de)

**Einleitung:**

Verletzungen des vorderen Kreuzbands (VKB) treten häufig bei jungen, aktiven Menschen auf, und dessen Rekonstruktion ist nach wie vor Therapiestandard. In letzter Zeit hat die biologische Augmentation mit autologen Thrombozytenkonzentraten (APC) aufgrund ihres Potenzials zur Verbesserung der Transplantatheilung an Interesse gewonnen. Diese Metaanalyse untersuchte, ob die Verwendung von APC die Patient-Related Outcome Measures (PROMs) verbessert und Komplikationen nach einer VKB-Rekonstruktion reduziert.

**Methoden:**

Es wurden randomisierte kontrollierte Studien (RCT) einbezogen, in denen die Standard-VKB-Rekonstruktion mit APC-verstärkten Verfahren verglichen wurde. Die in Frage kommenden Artikel wurden von Fachkollegen begutachtet und in englischer, deutscher, italienischer, französischer oder spanischer Sprache veröffentlicht. Im Juni 2025 wurde eine systematische Suche in PubMed, Web of Science und Embase durchgeführt. Die extrahierten Daten umfassten demografische Daten, Nachuntersuchungen und PROMs. Die methodische Qualität wurde mit dem Cochrane RoB2-Tool bewertet. Die Metaanalysen wurden mit Review Manager 5.3 unter Anwendung der inversen Varianz und der Mantel-Haenszel-Methode durchgeführt. Eine Subgruppenanalyse wurde für plättchenreiches Plasma (PRP) durchgeführt.

**Ergebnisse:**

Insgesamt erfüllten 14 RCTs mit 869 Patienten die Einschlusskriterien. Die methodische Qualität war akzeptabel, für keine Studie wurde ein hohes Verzerrungspotenzial festgestellt. Die durchschnittliche Nachbeobachtungszeit betrug 12,2 ± 7,4 Monate. Die Ausgangsmerkmale und PROMs waren zwischen den Gruppen vergleichbar. Die Verwendung einer APC-Augmentation reduzierte die vordere Tibialaxität signifikant, aber die Effektstärke lag unterhalb der klinischen Schwelle. Es wurden keine signifikanten Unterschiede bei den PROMs oder den Revisionsraten beobachtet.

**Schlussfolgerung:**

Der derzeitige Stand der Evidenz Level I spricht nicht für eine APC-Augmentation als Mittel zur Verbesserung der kurzfristigen Ergebnisse oder zur Verringerung von Komplikationen nach einer VKB-Rekonstruktion; jedoch rechtfertigen ihr Sicherheitsprofil und ihre biologische Begründung weitere hochwertige RCTs mit standardisierten Protokollen und längerer Nachbeobachtungszeit.

## Introduction

Anterior cruciate ligament (ACL) injuries are common, functionally debilitating, and particularly affect athletes and young active individuals [1, 2]. The incidence in the general population has been reported at 60–400 per 100,000 person-years, corresponding to 100,000–200,000 cases among athletes annually, with a 2.3% yearly increase over the past 2 decades [3, 4]. A higher risk is reported in individuals participating in pivoting sports and high-impact physical activities [5, 6]. The ACL stabilizes anterior tibial translation and rotational loads [5, 7]. Surgical reconstruction is particularly recommended for young, active and sportive patients aiming to return to high-level physical activity and for those patients who experience symptoms of persistent instability [8–11]. Despite refined surgical techniques, fixation devices and structured rehabilitation, complications remain, including graft failure, residual instability, pain and osteoarthritis [12, 13]. Successful reconstruction relies on ligamentization, the process of biological integration and remodelling of the graft [14, 15]. Reconstruction techniques use mechanical fixation systems, including screws, suspensory devices, staples, anchors and pins [16–18]. Healing after implantation depends on host biology, yet graft maturation may remain suboptimal during the early postoperative phase [19, 20]. This has prompted exploration of biological augmentation strategies. Among them, autologous platelet concentrates (APC), particularly platelet-rich plasma (PRP), have gained prominence [21–23]. The PRP is obtained from autologous blood and delivers a high concentration of platelets, releasing bioactive molecules that regulate proliferation, angiogenesis and extracellular matrix synthesis [24, 25]. These effects are mediated by platelet-derived growth factor (PDGF), transforming growth factor-beta (TGF-β), vascular endothelial growth factor (VEGF) and insulin-like growth factor 1 (IGF-1) [26, 27]. Preclinical data suggest that APCs accelerate fibroblast proliferation, neovascularization and collagen organization at the graft-bone interface [28, 29].

Clinical findings, however, remain inconsistent. Some studies reported improved postoperative pain and patient-reported outcome measures (PROMs), while others found no significant differences in function, stability or revision rates [29–32]. The evidence is weakened by methodological heterogeneity in design, PRP preparation and outcome reporting. Consequently, systematic reviews have often reached inconclusive results, with recommendations limited by low certainty and poor generalizability.

Given the contradictory literature, a meta-analysis of randomized controlled trials is warranted to clarify whether APC augmentation yields clinically meaningful improvements in ACL reconstruction. The present meta-analysis therefore investigated the efficacy of APCs by analyzing PROMs and complication rates, with a subgroup analysis focused on PRP.

## Methods

### Eligibility criteria

All the randomized controlled trials (RCTs) comparing ACL reconstruction versus APC-augmented ACL reconstruction were accessed. Only studies published in peer-reviewed journals were considered. According to the authors’ language capabilities, articles in English, German, Italian, French, and Spanish were eligible. Only studies with a level I evidence, according to the Oxford Centre of Evidence-Based Medicine [33], were considered. Reviews, opinions, letters and editorials were not considered. Animal, in vitro, biomechanics, computational, and cadaveric studies were not eligible.

### Search strategy

This study was conducted according to the 2020 Preferred Reporting Items for Systematic Reviews and Meta-Analyses (PRISMA) guidelines [34] and the recommendations of the Cochrane Handbook for Systematic Reviews of Interventions [35]. The PICOD algorithm was preliminarily established:

- P (problem): ACL tears,
- I (intervention): ACL reconstruction,
- C (comparison): APC-augmented ACL reconstruction,
- O (outcomes): PROMs and complications,
- D (design): RCT.

In June 2025, the following databases were accessed: PubMed, Web of Science, and Embase. No time constraint was set for the search. The Medical Subject Headings (MeSH) used for the database search are reported in the Appendix. No additional filters were used in the database search.

### Selection and data collection

The database search was independently conducted by two of the authors (N.J. and M.J.). All identified titles were manually screened and abstracts were reviewed when deemed relevant. Full texts were obtained for abstracts that aligned with the study topic; articles without accessible full texts were excluded. Additionally, the reference lists of included full-text articles were examined for further relevant studies. Any disagreements were discussed and resolved by the authors and if consensus could not be reached, a third senior author (F.M.) made the final decision.

### Data items

The following data were extracted at baseline: author, year of publication and journal, length of follow-up, number of patients in each treatment arm with related sex, mean age and mean BMI (kg/m^2^). Data concerning the following PROMs were collected at baseline and the last follow-up: visual analogue scale (VAS), Lysholm knee scoring scale [36], International Knee Documentation Committee (IKDC) [37] and knee injury and osteoarthritis outcome score (KOOS) [38]. The minimum clinically important difference (MCID) for the VAS was 2.7/10, 10/100 for the Lysholm score, 15/100 for the IKDC, and 10/200 for the KOOS [39–41]. The instrumental laxity evaluation performed with an arthrometer was referred to as KT-1000 and KT-2000 (MEDmetric Corp, San Diego, CA, USA). Both devices reproduce a linear force of 134 N to evaluate the knee laxity. These devices have been validated by other studies [42, 43]. The minimum detectable change (MDC) for KT-1000 and GNRB arthrometers ranges from 1.29–3.94 mm [44, 45]. Data from the following complications were also collected: infections, failures and revision surgery. Data were extracted in Microsoft Office Excel version 16.72 (Microsoft Corporation, Redmond, WA, USA).

### Methodological quality assessment and quality of the recommendations

The risk of bias was assessed according to the guidelines in the Cochrane Handbook for Systematic Reviews of Interventions [46]. One reviewer (L.S.) evaluated the risk of bias in the included studies. The RCTs were assessed using the revised Risk of Bias assessment tool (RoB2) [47, 48] of the Cochrane tool for assessing risk of bias in randomized trials (RoB). The following endpoints were evaluated: bias arising from the randomization process, bias based on deviations from intended interventions, bias due to missing outcome data, bias in the measurement of the outcome and bias in the selection of the reported result.

### Synthesis methods

The main author (F.M.) performed the statistical analyses. Mean and standard deviation were used for descriptive statistics. To evaluate the improvement from the baseline to the last follow-up, the IBM SPSS software version 25 (International Business Machines Corporation, Armonk, NY, USA) was used. The mean difference (MD) was calculated with a 95% confidence interval (CI). The paired t‑test was performed with values of *p* < 0.05 considered statistically significant. The meta-analyses were conducted using the software Review Manager 5.3 (The Nordic Cochrane Collaboration, Copenhagen, Denmark). Mean difference and standard deviation were used for descriptive statistics. The T‑test was performed to assess baseline comparability, with values of *p* > 0.1 considered satisfactory. The inverse variance method with mean difference (MD) effect measure was used for continuous data. The Mantel-Haenszel method with odds ratio (OR) effect measure was used for binary data. The CI was set at 95% in all the comparisons. Heterogeneity was evaluated through Higgins‑I^2^ and χ^2^ tests. If P_χ2_ > 0.05, no statistically significant heterogeneity was found. If P_χ2_ < 0.05, the heterogeneity was assessed following the values of the Higgins‑I^2^: low (< 30%), moderate (30–60%), and high (> 60%). A fixed effect model was set as default. If high heterogeneity was detected, a random model effect was used. Overall values of *p* < 0.05 were considered statistically significant.

## Results

### Study selection

**Fig. 1:**
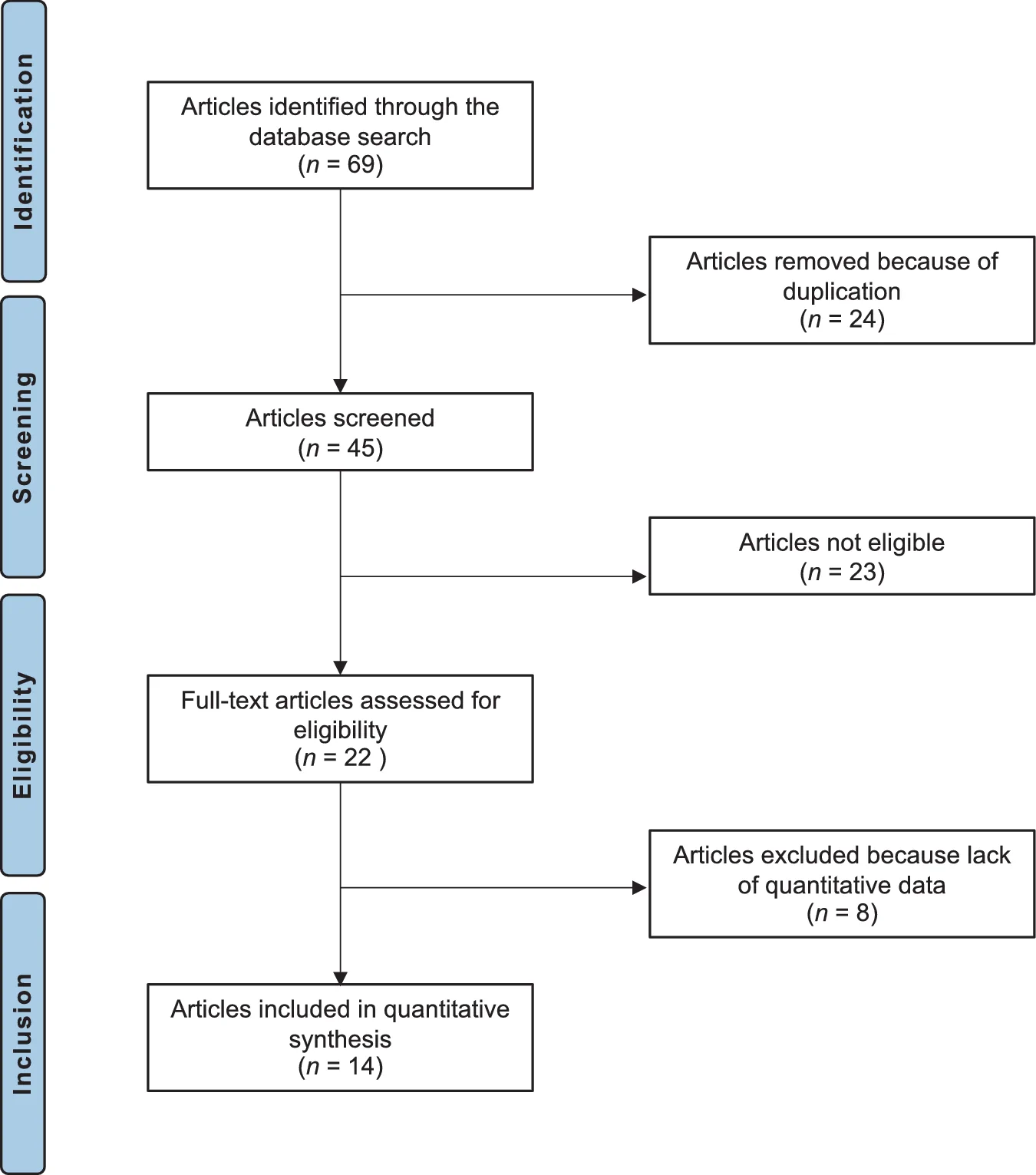
PRISMA flow chart of the literature search

An extensive search yielded 69 potentially relevant articles. After removing 24 duplicates, 45 records underwent title and abstract screening, with 23 excluded for reasons such as inappropriate study type or design (animal, cadaveric, biomechanical or nonclinical, *N* = 9), absence of a relevant control group (*N* = 6), or language limitations (*N* = 3). The remaining 22 articles were assessed in full, and 8 were excluded due to insufficient quantitative outcome data. Ultimately, 14 RCTs met the inclusion criteria. The selection process is shown in Fig. 1.

### Methodological quality assessment

**Fig. 2:**
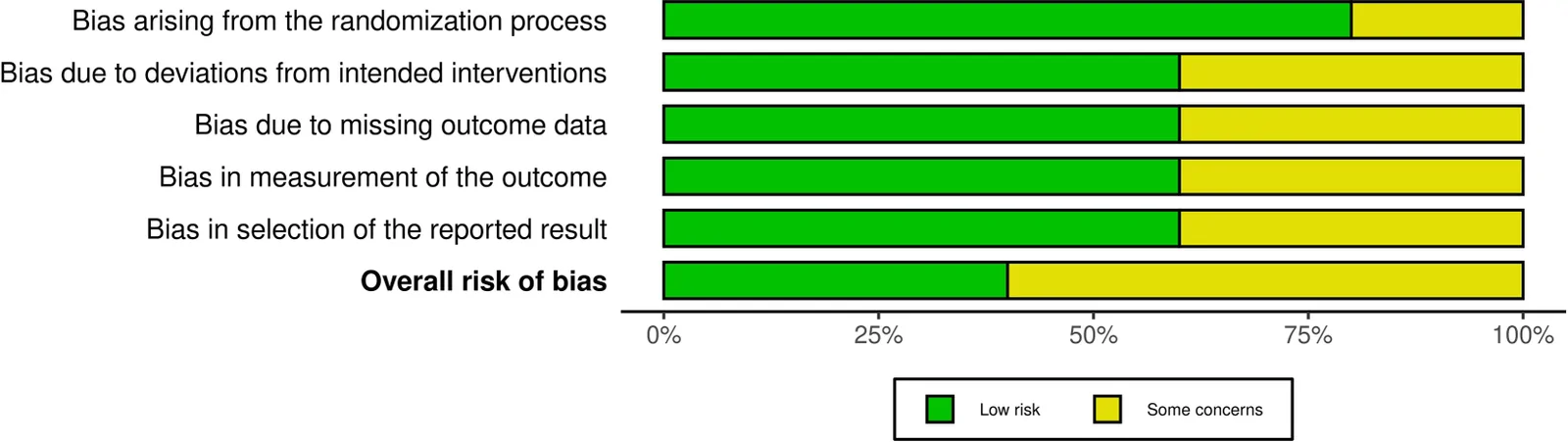
The RoB2 of the RCTs included in this study

All 14 studies were RCTs and assessed with the Cochrane RoB2 tool. Most trials adequately described the randomization process, with 64% (9/14) rated as low risk, while 5 raised some concerns due to unclear allocation or sequence generation, 3 studies raised concerns regarding protocol adherence or lack of blinding and 4 (29%) were due to incomplete follow-up or unclear handling of missing data. Outcome measurement was consistently robust with only one trial raising concerns and selective reporting risk was generally low. Overall, six studies were rated as having a low risk of bias and eight were rated as having some concerns but none were judged at high risk. These findings indicate acceptable methodological quality with minor limitations, mainly related to randomization and data completeness (Fig. 2).

### Study characteristics and results of individual studies

A total of 869 patients (869 knees) were included and 25% (215 of 869 patients) were women. The mean length of the follow-up was 12.2 ± 7.4 months. The mean age was 28.3 ± 4.2 years and the mean BMI was 25.2 ± 0.6 kg/m^2^. The general characteristics and demographics of the included studies are presented in Table 1.

**Table 1:** Generalities and patient baseline of the included studies

Author and year	Journal	Last follow-up (months)	Treatment	Patients (*n*)	Mean age (years)	Women (*n*)	Graft type
Azcarate et al. [49]	*Injury*	24.3	PRP and Leukocytes	50	26.1	10	Patellar tendon
			PRGF	50	27.4	7	
			Control	50	26.1	12	
Cervellin et al. [50]	*Knee Surg Sports Traumatol Arthrosc*	12	PRP	20	22.7	0	BPTB
			Control	20	22.9	0	
De Almeida et al. [51]	*Am J Sports Med*	6	PRP	12	25.8	2	BPTB
			Control	15	23.1	1	
Figueroa et al. [52]	*Arthroscopy*	6	PRP-ACP	30	26.8	12	Hamstring
			Control	20	23.6	5	
Gong et al. [29]	*Indian J Orthop*	12	PRP	30	33.5	12	Hamstring
			Control	30	34.9	9	
Kumar et al. [53]	*Cureus*	3	PRP	35	28.3	13	Hamstring
			Control	35	29.7	12	
Mirzatolooei et al. [54]	*Bone Joint J*	6	PRP	23	26.4	3	Hamstring
			Control	23	26.9	1	
Nin et al. [55]	*Arthroscopy*	24	PDGF	50	26.1	10	BPTB
			Control	50	26.6	12	
Starantzis et al. [56]	*J Sports Med (Hindawi Publ Corp)*	14.2	PRP	25	29.4	6	Hamstring
			Control	26	31.3	7	
Vadala et al. [57]	*Knee Surg Sports Traumatol Arthrosc*	14.7	PRP	20	33.75	0	Hamstring
			Control	20		0	
Ventura et al. [58]	*J Orthop Traumatol*	6	PRP	10	36.6	1	Hamstring
			Control	10	20.2	1	
Vogrin et al., [59]	*Wien Klin Wochenschr*	6	PLG	22	35.4	9	Hamstring
			Control	23	33.0	6	
Walters et al. [60]	*Am J Sports Med*	24	PRP	27	30.0	17	BPTB
			Control	23		11	
Ye et al. [61]	*JAMA Netw Open*	12	PRP	60	28.0	17	Hamstring
			Control	60	30.0	19	

*PRP* platelet-rich plasma; *PRGF* Plasma Rich in Growth Factors; *ACP* Autologous Conditioned Plasma; *PLG* Platelet Lysate Gel; *PDGF* Platelet-Derived Growth Factor; *BPTB* Bone-Patellar Tendon-Bone; *Control* Control group

### Baseline comparability

The mean age and BMI, number of women, mean arthrometer laxity and PROMs (Lysholm, VAS, IKDC) were comparable between the two groups (Table 2).

**Table 2:** Baseline comparability of patient characteristics between the two groups

Endpoint	ACL (*N* = 405)	ACL and APC (*N* = 464)	*P*
Mean age (years)	27.4 ± 4.5	29.1 ± 4.0	0.3
Women (%)	24% (96 of 405)	26% (119 of 464)	0.6
Mean BMI (kg/m^2^)	25.1 ± 0.5	25.3 ± 0.8	0.6
IKDC	48.4 ± 16.8	48.0 ± 18.1	0.9
Lysholm	60.6 ± 7.6	61.7 ± 7.7	0.8
VAS	5.6 ± 2.5	4.6 ± 4.8	0.8
Mean KT-1000	7.4 ± 2.2	7.0 ± 2.3	0.8

*ACL* Anterior Cruciate Ligament; *APC* Autologous Platelet Concentrate; *BMI* Body Mass Index; *IKDC* International Knee Documentation Committee; *VAS* Visual Analogue Scale; *KT-1000* Instrumented anterior laxity measurement

### Synthesis of results

**Fig. 3:**
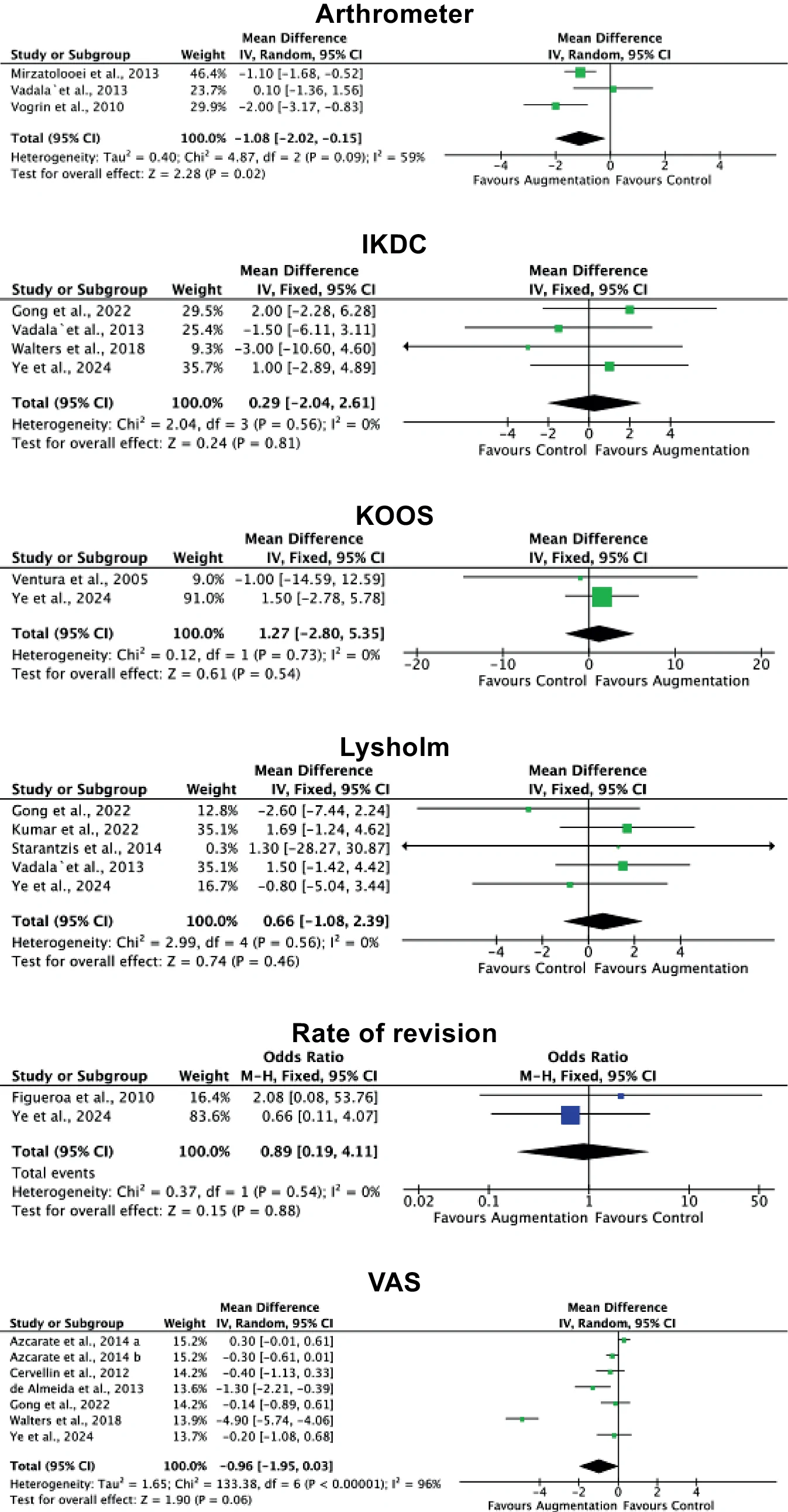
Results of the meta-analyses. Mean differences and 95% CI are shown for arthrometer-measured anterior tibial translation, IKDC, KOOS, Lysholm, VAS and OR for revision rates. *APC* autologous platelet concentrate, *ACL* anterior cruciate ligament, *IKDC* International Knee Documentation Committee, *KOOS* knee injury and osteoarthritis outcome score, *VAS* visual analogue scale, *MD* mean difference, *OR* odds ratio, *CI* confidence interval

The combined group evidenced a statistically lower laxity at the arthrometer (MD −1.08; 95% CI −2.02 to −0.15; *P* = 0.02). There was no statistically significant difference between the two groups in IKDC (*p* = 0.8), Lysholm (*p* = 0.5), VAS (*p* = 0.06) and KOOS (*p* = 0.5). No difference was evidenced in the rate of revisions. The forest plots of the meta-comparisons are presented in Fig. 3.

### Subgroup analysis

The subgroup PRP evidenced similar results. The combined group (ACL augmented with PRP) did not show any superiority in IKDC (MD 0.29; 95% CI −2.04–2.61; *p* = 0.8), Lysholm (MD 0.66; 95% CI −1.08–2.39; *p* = 0.5), VAS (MD −1.38; 95% CI −3.13–0.36; *p* = 0.1), arthrometer (MD −0.69; 95% CI −1.81–0.42; *p* = 0.2), KOOS (MD 1.27; 95% CI −2.80–5.35; *p* = 0.5), and revision (OR 0.66; 95% CI 0.11–4.07; *p* = 0.7).

## Discussion

According to the findings of the present meta-analysis, level I evidence indicates that augmenting ACL reconstruction with autologous platelet concentrates (APC) does not yield meaningful short-term improvements in patient-reported outcome measures (PROMs) or complication rates. Complications were rare in both the treatment and control groups. Although a statistically significant reduction in anterior tibial laxity was detected in the APC group, the magnitude of the difference fell below the threshold for clinical relevance. These data suggest that while the biological rationale for platelet-rich plasma (PRP) is compelling, its effects do not consistently translate into early clinical benefit following ACL reconstruction.

Given its concentration of growth factors, including PDGF, TGF‑β, VEGF, and IGF‑1, PRP has attracted considerable interest in orthopedics, which is believed to stimulate cell proliferation, promote angiogenesis and enhance collagen deposition [62, 63]. Within the context of ACL reconstruction, these properties could theoretically accelerate graft maturation and integration [64, 65]. Several imaging-based investigations have supported this biological hypothesis. Radice et al. [66] demonstrated a shortening of graft remodelling time on MRI, with complete homogeneity observed at 179 days in the PRP group compared with 369 days in the control group, suggesting nearly a halved maturation time. Similarly, Ruprecht et al. [67] reported reduced edema and increased vascular concentration at the tibial tunnel within 2.5 months of PRP administration, while Vogrin et al. [59] described higher vascularization at the osteoligamentous interface after 4–6 weeks. In a longer follow-up study, Seijas et al. [68] reported higher stages of remodelling on postoperative MRI at 4, 6 and 12 months after intra-articular administration of PRP, injected percutaneously into the suprapatellar space, following the portal suture used for ACL reconstruction.

In contrast to these promising results, other studies have reported no significant benefits [54, 57, 69]. Mirzatolooei et al. [54] reported no reduction in tunnel enlargement or laxity on CT or clinical testing at 12 weeks and Orrego et al. [69] similarly failed to demonstrate differences in graft-bone interface appearance 6 months postoperatively, despite intraoperative PRP administration. These discordant findings underline the variability of outcomes across trials.

The quantitative synthesis of arthrometer-measured anterior tibial translation revealed a between-group difference of 1.08 mm, which is below both the minimal detectable change and clinically accepted thresholds for pathological laxity [44, 45, 70]. As validated cut-offs for clinical insufficiency typically require side-to-side differences of at least 3–5 mm, the observed improvement likely represents statistical variation rather than biomechanical superiority. Importantly, PROMs such as KOOS, IKDC, Lysholm, and VAS did not show significant differences between groups. These findings are in line with some previous studies [55, 71, 72]. Magnussen et al. [72] concluded that despite biological plausibility, the use of PRP in ACL reconstruction did not improve functional outcomes or reduce the risk of revision 2 years after surgery. Similarly, Vadala et al. [57] found no differences between groups using physical examination and the evaluation scales employed. Nin et al. [55] found no statistically significant differences between the groups in clinical evaluation scores, inflammatory parameters or MRI appearance of the graft. These results emphasize the discrepancy between promising imaging outcomes and the absence of patient-perceived benefits.

The analysis also confirmed the safety profile of APCs in ACL reconstruction. No significant differences were observed in infection rates, failures or revisions between groups, in line with prior safety evaluations of PRP in musculoskeletal interventions [73, 74]. A retrospective comparison of 100 patients also documented no increase in complications with PRP augmentation [72]. The most frequently encountered complications remain rare, including tunnel malposition, stiffness, infection or patellar fracture; however, their incidence is insufficient to enable meaningful quantitative pooling [9, 75–78]. Although the present meta-analysis focused on primary ACL reconstructions using autografts, it is reasonable to consider whether APCs might provide benefits in more biologically demanding situations. While current high-level evidence does not support their routine use, some studies have explored potential advantages in specific subgroups. Lin et al. [79] reported that combining BMAC with PRP resulted in greater improvements in knee laxity and more favorable MRI findings of graft-bone interface healing, although functional outcomes remained comparable to controls. Similarly, Solomon et al. [80] observed reduced tunnel widening and enhanced graft maturation when PRP was combined with demineralized bone matrix during ACL reconstruction. Another randomized study found improved early radiological and functional outcomes following PRP augmentation [81]. Taken together with preclinical evidence demonstrating enhanced angiogenesis, cell proliferation and collagen organization with platelet-derived factors, these results suggest that APCs could be of interest in revision surgery, allograft procedures, or in patients with compromised healing capacity [82]; however, these findings remain preliminary; they provide a rationale for future trials investigating the potential applications of APCs.

This study has several limitations. Most included RCTs had relatively short follow-up durations, typically between 6 and 12 months, which limits insights into long-term effects. Furthermore, the lack of blinding in most studies introduces a potential risk of bias. There was considerable heterogeneity in the PRP preparation methods and application protocols, making comparisons difficult. Additionally, PROMs varied across studies and may not fully capture biologically relevant changes. Imaging-based outcomes, such as graft signal intensity or tunnel widening, were not consistently reported and could not be included in the meta-analysis. The low event rate constrained the analysis of complications. Consequently, we were unable to statistically compare the complication rates between the isolated ACL reconstruction group and the group receiving autologous platelet concentrates. Different PRP formulations (e.g., leukocyte-rich or leukocyte-poor preparations) were not analyzed separately, as most included trials did not specify leukocyte content or platelet concentration in sufficient detail and all PRP-based interventions were pooled within the APC category. These limitations restrict the ability to draw firm conclusions regarding the safety profile of the interventions. They highlight the need for larger, well-powered studies with standardized and systematic reporting of complications to facilitate more robust analyses in the future.

## Conclusion

The current level of evidence does not recommend the use of APC augmentation during ACL reconstruction. While APC augmentation during ACL reconstruction does not significantly improve short-term functional outcomes or complication rates, its safety profile and biological potential warrant further investigation. High-quality RCTs with standardized PRP protocols, more extended follow-up periods and integration of imaging and functional endpoints are needed to clarify its clinical relevance.

## Supplementary Information


Supplementary material 1


## Data Availability

The datasets generated and/or analyzed during the current study are available throughout the manuscript.

## Abbreviations

ACL: Anterior cruciate ligament
APCs: Autologous platelet concentrates
IGF-1: Insulin-like growth factor-1
IKDC: International Knee Documentation Committee
KOOS: Knee Injury and Osteoarthritis Outcome Score
MCID: Minimum clinically important difference
PDGF: Platelet-derived growth factor
PRP: Platelet-rich plasma
RCTs: Randomised controlled trials
TGF-β: Transforming growth factor-β
VAS: Visual Analogue Scale
VEGF: Vascular endothelial growth factor
