# Supplementary material for: Autologous platelet concentrate augmentation in anterior cruciate ligament reconstruction: a level I evidence-based meta-analysis
Source: Orthopadie (Heidelb). 2025 Nov 28;55(9):745–54. doi: 10.1007/s00132-025-04748-y (PMC13525064; doi:10.1007/s00132-025-04748-y)
Supplement: Supplementary file 1 [file 132_2025_4748_MOESM1_ESM.pdf]

Systematic literature search for:

Autologous platelet concentrates augmentation in anterior cruciate ligament reconstruction: a level I evidence-based meta-analysis

Concept 1: ACL Tears / ACL Reconstruction

Keywords:
ACL tear
ACL injury
anterior cruciate ligament rupture
anterior cruciate ligament reconstruction
ACL reconstruction

Mesh:
"Anterior Cruciate Ligament"[Mesh]
"Anterior Cruciate Ligament Reconstruction"[Mesh]

Concept 2: Intervention (Autologous Platelet Concentrates)

Keywords:
platelet-rich plasma (PRP)
autologous platelet concentrate
platelet concentrate
platelet gel
PRF (platelet-rich fibrin)

Mesh:
"Platelet-Rich Plasma"[Mesh]
"Blood Platelets"[Mesh]

Concept 3: Interest of the outcome (PROMs, complications)

Keywords:
PROMs
patient-reported outcome
VAS
visual analogue scale
IKDC
Lysholm
Tegner
subjective knee score
complications
adverse events
re-injury
reoperation
graft failure

Mesh:
"Patient Outcome Assessment"[Mesh]
"Patient Reported Outcome Measures"[Mesh]

Concept 1:

ACL tear OR ACL injury OR anterior cruciate ligament rupture OR anterior cruciate ligament reconstruction OR ACL reconstruction OR "Anterior Cruciate Ligament"[Mesh] OR "Anterior Cruciate Ligament Reconstruction"[Mesh]

AND

Concept 2:

platelet-rich plasma (PRP) OR autologous platelet concentrate OR platelet concentrate OR platelet gel OR PRF (platelet-rich fibrin) OR "Platelet-Rich Plasma"[Mesh] OR "Blood Platelets"[Mesh]

AND

Concept 3:

PROMs OR patient-reported outcome OR VAS OR visual analogue scale OR IKDC OR Lysholm OR Tegner OR subjective knee score OR complications OR adverse events OR re-injury OR reoperation OR graft failure OR "Patient Outcome Assessment"[Mesh] OR "Patient Reported Outcome Measures"[Mesh]

NOT

hip

Summary of the Search

(((ACL tear OR ACL injury OR anterior cruciate ligament rupture OR anterior cruciate ligament reconstruction OR ACL reconstruction OR "Anterior Cruciate Ligament"[Mesh] OR "Anterior Cruciate Ligament Reconstruction"[Mesh]) AND (platelet-rich plasma (PRP) OR autologous platelet concentrate OR platelet concentrate OR platelet gel OR PRF (platelet-rich fibrin) OR "Platelet-Rich Plasma"[Mesh] OR "Blood Platelets"[Mesh])) AND (PROMs OR patient-reported outcome OR VAS OR visual analogue scale OR IKDC OR Lysholm OR Tegner OR subjective knee score OR complications OR adverse events OR re-injury OR reoperation OR graft failure OR "Patient Outcome Assessment"[Mesh] OR "Patient Reported Outcome Measures"[Mesh])) NOT (hip)
